# Ultra-Sensitive Biosensors for Medical Applications Based on Nanomechanics: From Detection of Synthetic Biomolecules to Analysis of Sepsis in Pediatric Patients

**DOI:** 10.3390/bios15040217

**Published:** 2025-03-28

**Authors:** François Huber, Hans Peter Lang, Andrea Marten, Julia Anna Bielicki, Ernst Meyer, Christoph Gerber

**Affiliations:** 1 Swiss Nanoscience Institute (SNI), Department of Physics, University of Basel, CH-4056 Basel, Switzerland; hans-peter.lang@unibas.ch (H.P.L.); ernst.meyer@unibas.ch (E.M.); 2 University Children’s Hospital Basel (UKBB), Department of Medicine, University of Basel, CH-4056 Basel, Switzerland; andrea.marten@ukbb.ch (A.M.); juliaanna.bielicki@ukbb.ch (J.A.B.)

**Keywords:** bacteremia, sepsis, nanomechanical biosensors, cantilevers, bacterial infections, total RNA, rapid sensitive diagnostics, cancer diagnostics, biomolecular interactions, DNA hybridization

## Abstract

Recent advancements in nanomechanical microcantilever biosensors open new possibilities for clinical applications, permitting precise analysis of molecular interactions. The technology enables tracking gene expression, molecular conformational changes, antibody binding and antibiotic resistance. In particular, hybridization of DNA or RNA extracted from biopsies and whole blood from patients has led to significant advancements in diagnostics of critical medical conditions, e.g., cancer, bacteraemia and sepsis, utilizing rapid, sensitive, and label-free detection. Direct diagnosis from patient samples is a decisive advantage over competitive methods circumventing elaborate and time-consuming purification, amplification and cultivation procedures prior to analysis. Here, recent developments are presented from simple DNA hybridization of synthesized oligonucleotides to RNA material obtained from patients’ blood samples, highlighting technological advancements in diagnostic applications, such as detection of pathogens and disease biomarkers. We envisage our method to be a significant input to rapid, early and sensitive diagnosis directly from patients’ blood without requirements for amplification or cultivation. This would represent a paradigm shift in diagnostics, as no competing method currently exists.

## 1. Introduction

**Figure 1:**
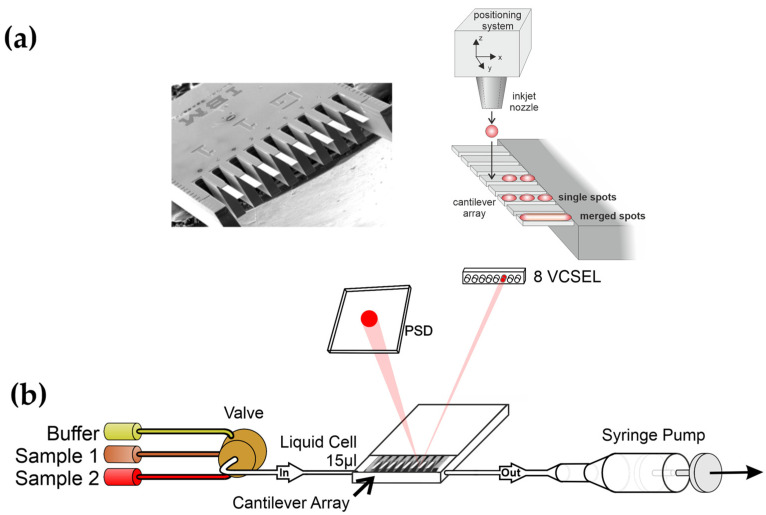
(**a**) Eight gold-coated silicon microcantilevers 500 µm long and 600 nm thick arranged in an array with a pitch of 250 µm is shown being functionalized using an inkjet spotter (adapted from [1]). (**b**) Schematic of experimental setup. Different liquid samples can be applied sequentially with the help of a syringe pump and a multiway valve (adapted from [10]).

**Figure 2:**
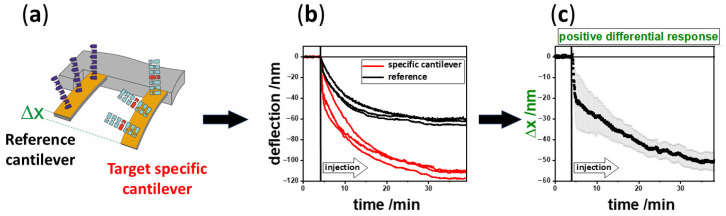
Schematic of a nanomechanical biosensor experiment (adapted from [10]). (**a**) For simplicity we only show 2 out of 8 sensors. Target specific gold coated cantilevers are functionalized with thiolated DNA probes to corresponding targets in liquid samples. Internal references are mandatory for differential readout to reduce thermal drift and nonspecific binding artifacts (**b**) Specific responses are shown in red and references in black. (**c**) Averaged differential signal (differences in responses of red specific and black reference cantilevers) with corresponding standard deviation.

There is a need for fast and reliable diagnostic devices that are compact and affordable to implement in clinical settings or doctoral offices. Thus, rapid diagnosis is decisive for an early treatment to the benefit of patients. The system we use is based on 8 nanomechanical microcantilevers in an array format for surface stress detection based on scanning probe microscopy beam deflection. In principle, the molecular interaction between an analyte and receptor on a surface is transduced into a nanomechanical motion of a microcantilever, denoted as static deflection mode. The receptors are immobilized using inkjet spotting [1] as a layer on one of its surfaces (Figure 1). The array is mounted in a microfluidic cell and exposed to a liquid sample. A multiway valve allows for switching between buffer and different samples. Microcantilevers will bend due to biomolecular interactions and bending is determined with a precision of 0.1 nm via a multiplexed laser beam deflection readout. Biomolecular interactions produce a change in surface stress, due to changes in molecular configuration and intermolecular crowding [2,3], resulting in bending of the cantilever. The molecular mechanism of surface stress-based adsorption has been investigated by molecular dynamic simulations [4,5]. Others used a percolation model [6] to explain surface stress changes at microcantilever surfaces. Surface stress changes can also be directly observed by AFM [7]. In Figure 2 we show a schematic of binding via DNA/DNA hybridization [8,9].

### 1.1. Hybridization of Synthetic Oligonucleotides

Initial experiments were performed using chemically synthesized unlabeled DNA target sequences. It was found that responses are target specific and concentration dependent. The detection process is predominantly based on steric hindrance effects resulting in nanomechanical bending. Experiments with different 3’ and 5’ overhang extensions generally reduced the signal indicating that surface stress changes at the binding site of the oligonucleotides are mainly responsible for the development of the nanomechanical bending response of the microcantilever sensor. Moreover, microcantilevers have been applied to examine mechanically the thermodynamics of biomolecular interactions [11], such as the equilibrium dissociation constant (K^−1^) and the change in Gibbs free energy (ΔG) for DNA/DNA interactions occurring on the cantilever surface [12,13].

For a deeper understanding of protein expression, the technique has been applied to the binding of transcription factors [14], which play an important role in the initial steps of protein expression. They bind in a sequence specific way to double stranded DNA initiating transcription of mRNA. Transcription factors SP1 and NF-κB were investigated using specific recognition sites. Here, hairpin loop forming [15] DNA oligonucleotides comprising a sequence specific site in the stem for transcription factor binding, were chemisorbed to gold-coated cantilevers via a terminal thiol group. By applying a range of transcription factor concentrations from 100 to 400 nM we measured a concentration dependence of the bending signal. Furthermore, recognition sites can be alternately used as references for each transcription factor, emphasizing the specificity of transcription factor binding.

### 1.2. Antigen Detection with Antibodies

As a medical application, continuous label-free detection of creatine kinase and myoglobin (two cardiac disease biomarker proteins) was demonstrated using a microcantilever biosensor array functionalized with anti-creatine kinase and anti-myoglobin whole antibodies [16]. Protein biomarker recognition is detected via changes in surface stress generated by antigen–antibody binding [17]. All experiments included reference cantilevers to prevent signals from undesired effects, such as nonspecific binding. The method achieved a sensitivity below 20 µg mL^−1^ myoglobin. Detection of myoglobin and creatine kinase were detected independently in an unspecific protein background using matching antibodies. The array format allows for the use of up to seven different antigen–antibody reactions with an additional in situ reference simultaneously. The main application lies in early and rapid diagnosis of acute myocardial infarction. Detection of bacterial pathogens require a rapid response for adequate treatment of sepsis patients. Here, lipopolysaccharide antibodies are used to detect specific endotoxins to identify bacterial species responsible for the sepsis [18].

In the field of immunosensors, microcantilever arrays showed a performance comparable to surface plasmon resonance [19]. Here, single-chain Fv (scFv) antibody fragments were used as receptor probes. For direct and oriented covalent attachment to gold-coated microcantilevers, a cysteine residue was introduced at the C terminus of a scFV thereby improving sensitivity significantly. The introduction of oriented scFv fragments as probes increased the sensitivity 1000-fold compared to whole antibodies.

### 1.3. Protein Conformation Changes

We applied the method to investigate changes in protein conformation [20] by functionalizing a cantilever surface with light sensitive enzymes that change their conformation upon exposure to light [21]. For this study, microcantilevers were functionalized with proteoliposomes containing bacteriorhodopsin to investigate conformational changes in membrane proteins. The key outcome was the detection of a light induced protein conformation variation due to the removal of the retinal, resulting in nanomechanical surface stress change. The response is quantitative and depends linearly on the amount of removed retinal. These results show the technique is capable to measure membrane protein-based receptor-ligand interactions and conformational changes.

### 1.4. Cancer Diagnostics

**Figure 3:**
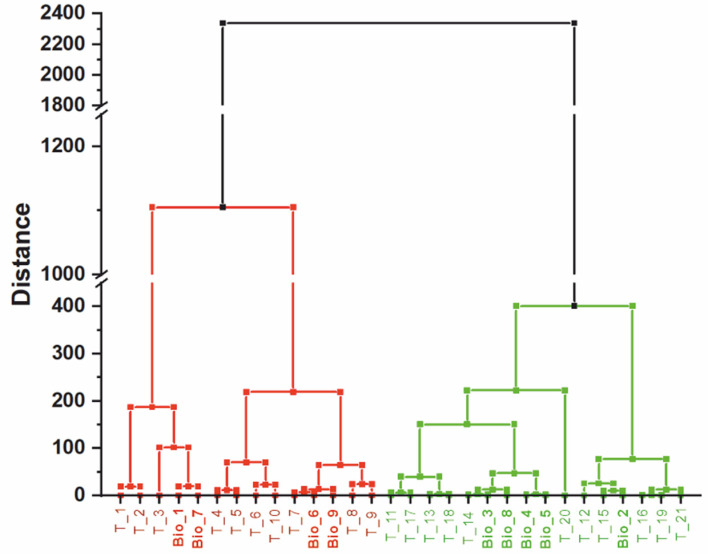
Hierarchical tree of a cluster analysis. 11 *BRAF^V600E^* positive (green branches, T_1–T_10), 11 *BRAF^V600E^* negative (red branches, T_11–T_21) tissue culture samples and 9 biopsies (Bio_1–Bio_9) are shown. The *BRAF^V600E^* positive biopsies 1, 6, 7, and 9 are indicated in bold red and the *BRAF^V600E^* negative 2, 3, 4, 5, and 8 biopsies are depicted in bold green. The method of sum of distances was used to calculate the Euclidian distances (adapted from [28]).

Our technique cannot only be applied to synthetic samples, but is currently taken to the clinic examining biopsies and blood samples from patients. Different microcantilever based studies address various types of cancers, e.g., breast, liver cancer and cancer biomarkers in general [22,23,24,25]. We focused on the detection of specific cancer markers from malignant melanoma, using the *BRAF* gene (gene B of Rapid Acceleration of Fibrosarcoma) [26] using RNA targets isolated by a simple nucleic acid extraction step from natural samples, such as tissue culture melanoma cells [27]. Additionally, we conducted a preliminary double-blind study with biopsies from malignant melanoma. We detected a single nucleotide polymorphism (SNP, *BRAF^V600E^*) in total RNA extracted from biopsies [28]. The study clearly distinguished *BRAF^V600E^* positive cells and biopsies from *BRAF^V600E^* negative samples (Figure 3). This analysis also helps in identifying the proper mutations, here *BRAF^V600E^*, for highly specific treatments (such as the BRAF inhibitor vemurafenib) that only target cancer cells and will not affect healthy cells and thereby improve a patient’s condition.

### 1.5. Sepsis Diagnostics

**Figure 4:**
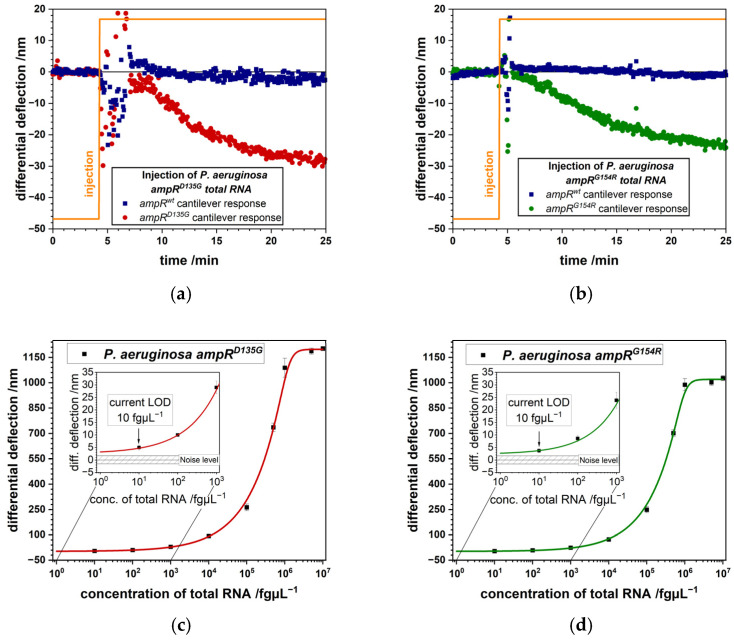
Differential microcantilever responses to flushing with 1 pg µL^−1^ total RNA: (**a**) differential signal (red) of mutation D135G and reference microcantilever. (**b**) differential signal (green) of mutation G154R and reference microcantilever. Signal size is 28 ± 3 nm at 1 pg µL^−1^ total bacterial RNA. Present limit of detection (LOD) for total RNA extracted from *P. aeruginosa ampR^D135G^* (**c**) and *ampR^G154R^* (**d**) strains, respectively. In the insets an LOD of 10 fg µL^−1^ is displayed as calculated from the logistic fit, which corresponds to 10 bacterial cells with a sensitivity of less than 2 nm (adapted from [39]).

**Figure 5:**
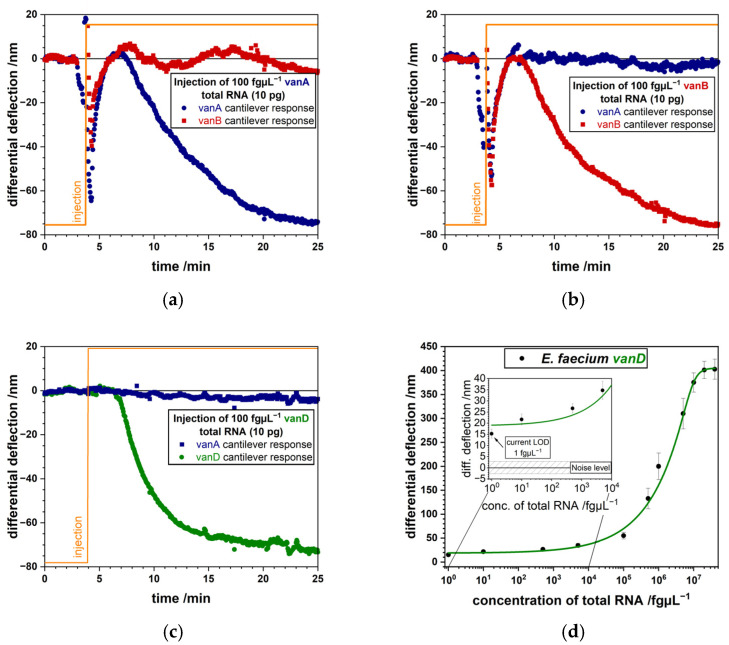
Injecting a total amount of 10 pg RNA at 100 fg µL^−1^ of vanA, vanB and vanD samples yields specific responses (adapted from [39]). (**a**) Signal of *vanA* (blue) coated microcantilevers upon injection of *vanA* producing *E. faecium* RNA versus *vanB* (red) coated microcantilevers. (**b**) Response of *vanB* (red) coated microcantilevers upon injection of *vanB* producing *E. faecium* RNA versus *vanA* (blue) coated microcantilevers. (**c**) Signal of *vanD* (green) coated microcantilevers upon injection of *vanD* producing *E. faecium* RNA versus *vanA* (blue) coated cantilevers. (**d**) Determining a limit of detection (LOD) for the *vanD* gene of *E. faecium*. 1 fg µL^−1^ to 40 ng µL^−1^ serial dilution of total RNA is measured. The inset shows an LOD of the method of 1 fg µL^−1^ as established from the logistic fit which corresponds to a single bacterial cell.

A big advantage is that the same nanomechanical biosensor platform can be used to examine gene transcription, translation and structural changes in enzymes. Particularly, sepsis diagnostics will benefit from such devices, because of the presence of bacterial pathogens and antibiotic resistances requires a quick adaptation of treatment strategies. K. E. Rudd et al. [29] in a study estimated there were 30 million infections and 11 million sepsis-related deaths worldwide in 2017. A more recent NIH study [30] published in 2023 shows a significant increase of mortality rate in sepsis related cases. Moreover, sepsis accounts for about 2.9 million worldwide deaths in children less than 5 years old. Therefore, fast and consistent diagnosis of the pathogens is of great importance [31]. The reference standard for blood stream infections is blood culture-based, but it has certain disadvantages such as a sensitivity of about 67.7% [32] and take considerable time to identify a bacterial species. Incubation time of blood cultures can be up to 48 h for it to turn positive, at that point subcultures are prepared to get single bacterial colonies. Many emerging technologies are under current investigation [33] to accelerate diagnostics of sepsis among them are nanomechanical biosensors [6,34,35,36,37]. Nanomechanical microcantilevers detected single nucleotide polymorphisms (SNPs) [38] and different genes connected to antibiotic resistance in Gram negative (*Pseudomonas aeruginosa*) and positive (*Enterococcus faecium*) bacteria, which represent common causes for multi drug resistant (MDR) infections [39,40]. Highly specific RNA capture probes for SNPs in different ampicillin resistances (*ampR^D135G^* or *ampR^G154R^*) or various vancomycin resistance genes (*vanA*, *vanB*, and *vanD*) were used which allowed us to identify the RNA of resistance markers in less than 5 min. Unprecedented sensitivity was achieved of less than ten bacterial cells, equivalent to 10 fg µL^−1^ bacterial RNA, for *ampR* SNPs (Figure 4) and 1 bacterial cell, corresponding to 1 fg µL^−1^ bacterial RNA, for *vanD* (Figure 5), as calculated from serial dilutions.

In this study, in clinics, frequently antibiotic resistance markers such as different SNPs in the *ampR* gene important for *ampC* overexpression [41] were investigated. We designed probes for surface modification covering the site of the SNP with significant overhangs up- and downstream. While these overhangs decrease the response due to steric hindrance, they do not interfere in a significant way with binding to the microcantilever. For the detection of whole genes, such as *vanA*, *vanB* or *vanD* we can choose a position to place the probe sequence and therefore large overhangs can be reduced to just one end of the RNA transcript. For that purpose, the location of the probe was selected to be at the 3′-end of the gene to improve sensitivity due to the close proximity of the binding site to the microcantilever surface [42]. Highly specific detection of *vanD* RNA was demonstrated at only 1 bacterial cell per milliliter fluid. Additionally, to the exceptional sensitivity and specificity, the method allows fast response times in the range of minutes. It took typically 20 min after sample injection to measure an individual point in the Langmuir plot. The limit of detection (LOD) is of great importance, because in some cases only low numbers of bacteria per mL fluid may be present in the early stages of a bacterial infection. The high sensitivity permits diagnosis directly from a patient’s specimen without culture, label-free and no amplification, based on a simple RNA extraction. Antibiotic resistance genes, such as *vanD* are detected in total RNA from 100 fg corresponding to just 1 bacterial cell. SNPs in the *ampR* gene are detected in 1 pg total RNA equivalent to 10 bacterial cells.

We apply total RNA from two different bacterial species showing different resistance mechanisms. AmpR^G154R^ ratio in total bacterial RNA compared to vanD is lower and therefore require a higher concentration of total RNA for detection. This explains the 10 fg/µL ampR^G154R^ to 1 fg/µL vanD concentration difference. The developments presented so far show the vast progress microcantilever biosensors have made in recent years. This progress paved the way for rapid and highly specific and sensitive detection of various diseases at an early stage. The current study extends the application of the method to important questions concerning sepsis in pediatric patients.

## 2. Materials and Methods

### 2.1. RNA Preparation from Blood

For performance assessment, we collected EDTA whole blood samples from children with positive and negative blood cultures as controls at the University Children’s Hospital Basel. Bacteria in the blood culture-positive material were identified using blood culture techniques [43]. For the total RNA isolation, the EDTA (ethylenediaminetetraacetic acid) whole blood samples were first stabilized with RNAprotect^®^ Bacteria Reagent (QIAGEN GmbH, Hilden, Germany) by mixing 1 volume of sample with 2 volumes of reagent, briefly incubating, centrifuging for 10 min at 5000× *g*, and storing the pellet at −80 °C. On the day of RNA extraction, the pellet was treated with TE buffer (MERCK) containing 15 mg/mL lysozyme (MERCK), and proteinase K (QIAGEN GmbH) to break down cell walls. Following the addition of RNeasy Lysis Buffer (QIAGEN GmbH) containing ß-mercaptoethanol (MERCK) and mechanical lysis using glass beads (MN glass beads Typ B 740,812.50, Macherey-Nagel, Düren, Germany), the supernatant was isolated and mixed with ethanol. The RNA was then purified using the RNeasy^®^ Mini Kit (QIAGEN GmbH) including DNase digestion, aliquoted, and the concentration was measured using Titertek-Berthold Colibri (Berthold Technologies GmbH & Co. KG, Bad Wildbad, Germany). Concentrations were in the range of 5 ng/µL to 500 ng/µL.

### 2.2. Biosensor Preparation

Microcantilever arrays were obtained from IBM Research (Rüschlikon, Switzerland). A procedure described previously [1] to functionalize microcantilevers with oligonucleotides was applied. The arrays were cleaned for 15 min with a piranha solution (30% H_2_O_2_:96% H_2_SO_4_ = 1:1, *v*/*v*) washed three times with water, finally rinsed with isopropanol and dried in air. The arrays were first coated with a 2 nm thick layer of titanium followed by 20 nm gold. The gold surface allows thiolated probe oligonucleotides to form a self-assembled monolayer (SAM) for highly specific and sensitive detection of target molecules.

The oligonucleotides presented in Table 1 for the detection of bacteria were used during all experiments. Microsynth AG (Balgach, Switzerland) provided the thiol modified oligonucleotides at a concentration of 100 µM without dithiothreitol (DTT). Oligonucleotides were diluted to 20 µM in a buffer containing 50 mM tri-ethyl-ammonium-acetate buffer (TEAA) obtained from Sigma-Aldrich (Buchs, Switzerland) and 1 mM tris(2-carboxyethyl) phosphine (TCEP) obtained from Sigma-Aldrich (Buchs, Switzerland) and high-performance liquid chromatography (HPLC) grade DEPC (0.1% diethylpyrocarbonate) treated water (BioConcept, Allschwil, Switzerland). A Microdrop inkjet printer (MDP705L, Microdrop Technologies, Norderstedt, Germany) was used to functionalize microcantilever arrays. Typically target specific probes, e.g., 16S-rRNA and reference probes were applied, subsequently arrays were incubated for one hour at room temperature. The array was placed in a measurement chamber with a volume of 15 µL filled with 0.03 × SSC (saline sodium citrate buffer, prepared using 20 × SSC from Sigma Aldrich).

20 nucleotide long oligonucleotide probes enabled improved hybridization, because probes shorter than 24 nucleotides form SAMs covalently attached by thiol groups [3]. A standardized bacteria negative blood reference was created by pooling total RNA samples from 15 bacteria negative adult individuals. The reference was used to establish a baseline in advance of patient sample injection.

### 2.3. Experimental Procedure

The chamber was placed in a temperature-controlled box. Bending of all eight microcantilevers was measured as described earlier with sub-nanometer accuracy [46]. All total RNA samples were diluted in 0.03 × SSC buffer which was used throughout the measurements. First, a baseline was established ahead of sample application by injecting total RNA extracted from pooled whole blood samples of bacteria negative individuals to minimize nonspecific influences from blood alone. Second, 100 pg µL^−1^ RNA from bacteria positive patients was injected. Last, buffer was used to purge sensors. We only use total bacterial RNA extracted from whole blood samples, as RNA from other cells was removed during RNA extraction. Experiments were conducted at 27 °C and with a flow rate of 20 µL/min for a total of 200 µL to 400 µL. We use freshly prepared sensors for single use in each measurement, therefore long-term performance stability is not an issue for the analysis.

## 3. Results

So far, we have demonstrated that nanomechanical biosensors can successfully be applied in the detection of biochemical reactions such as antigen detection, structural changes in proteins, transcription factor binding and DNA/RNA hybridization with unprecedented sensitivity and specificity, label free and rapid response. We have made the transition from synthetic materials, i.e., oligonucleotides, to actual patient samples from the clinic. The above presented achievements encompass cancer diagnostics based on biopsies and blood samples from adult patients. In the following experiments we address sepsis in pediatric patients. The swift detection of the bacterial pathogen, particularly in the initial stages is crucial to prevent inflammatory responses and organ failures. To avoid lengthy processes such as bacterial culturing, our method as pointed out above offers significant advantages. As part of this preliminary study, we obtained total RNA from EDTA whole blood samples from children with bacteremia confirmed through blood culture, which is the current diagnostic gold standard.

### 3.1. Detection of Bacterial Pathogens

**Figure 6:**
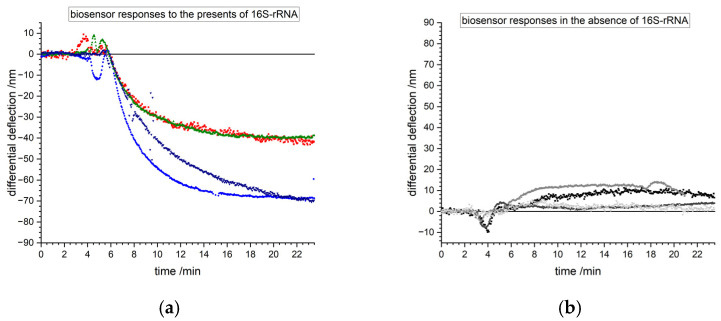
Examples of measurement curves from the injection of total RNA samples from different patients labeled in different colors. (**a**) Nanomechanical biosensors bend downwards, showing representing 16S-rRNA binding indicating a bacterial infection. (**b**) Sensors bend slightly upwards due to residues from the purification steps despite of absence of 16S-rRNA.

Initial experiments were conducted to show the feasibility of the method to identify the presence of bacterial pathogens using the prokaryote small ribosomal subunit 16S-rRNA as the target sequence. This subunit is evolutionary very well conserved among different bacterial species and therefore an ideal target for the detection of bacterial pathogens. Nanomechanical biosensors show complete opposite reactions in the presence or absence of 16S-rRNA (Figure 6). Binding of the target 16S-rRNA to the surface probes creates a compressive stress resulting in a downward bending of the cantilevers. The probe is designed to detect bacteria. In the absence of bacteria, no or only a small signal is observed due to unspecific adsorption. The small residual signal might appear as a drift in Figure 6b despite of differential measurements. It is actually the response to unspecific adsorption to the lower surface of the microcantilever. In Figure 6a such residual signals also occur but are suppressed by the much larger specific signal due to hybridization.

### 3.2. Comparison of Different Age Groups

**Figure 7:**
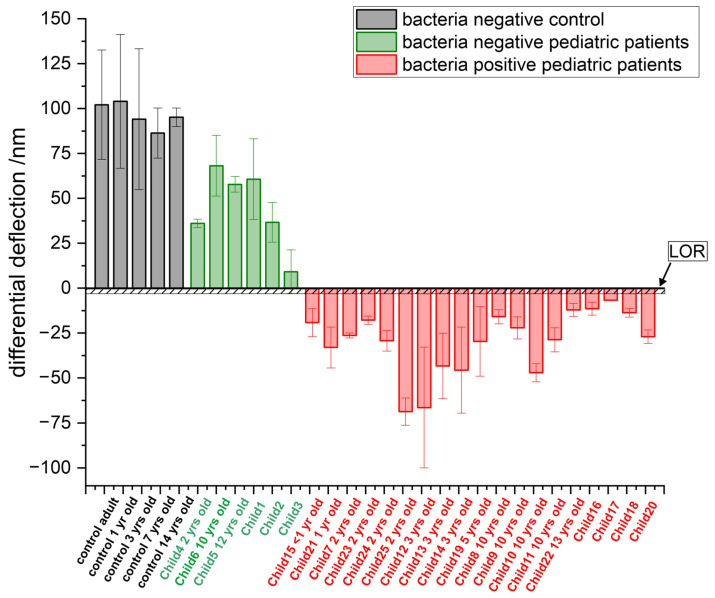
Sensor responses by age group. The first 5 grey bars compare blood samples from healthy individuals (controls), including an adult patient and pediatric patients of different age groups (1-year-old infant, 3, 7 and 14-years old children). The next 6 green bars (with a positive signal) show healthy pediatric patients, and the remaining 19 red bars (with a negative signal) are bacteria positive pediatric patients. Also shown is the level of rejection (LOR) which is around 3 nm, above which are considered bacteria negative.

Blood composition in children may vary with age [47] where changes in miRNA expression levels are observable. First, total RNA samples from healthy individuals are injected into the measurement chamber to create a stable baseline and exclude nonspecific contributions. To address the age issue, blood samples from a bacteria negative adult sample with children of different age groups (Figure 7) were analyzed to assess the influence of blood composition to the measurements. Bacteria negative patients show a positive differential deflection signal whereas bacteria positive pediatric patients show throughout a negative differential deflection signal, but no dependence on patients’ age was observed. All measured data clearly surpassed the limit of rejection (LOR). Bacteria negative (healthy) and bacteria positive (diseased) pediatric patients can be clearly identified by the sign of the sensor response.

### 3.3. Extended Study with 233 Total RNA Samples

To establish the above findings, we conducted a larger study with 233 total RNA samples from pediatric patients. The results were analyzed to calculate important key figures to evaluate the performance nanomechanical biosensors (Equations (1)–(4), adapted from [48]).

Sensitivity (SE) = TP/(TP + FN),(1)

Specificity (SP) = TN/(TN + FP),(2)

Positive predictive value (PPV) = TP/(TP + FP),(3)

Negative predictive value (NPV) = TN/(TN + FN),(4)

True positive (TP); True negative (TN);

False positive (FP); False negative (FN).

**Figure 8:**
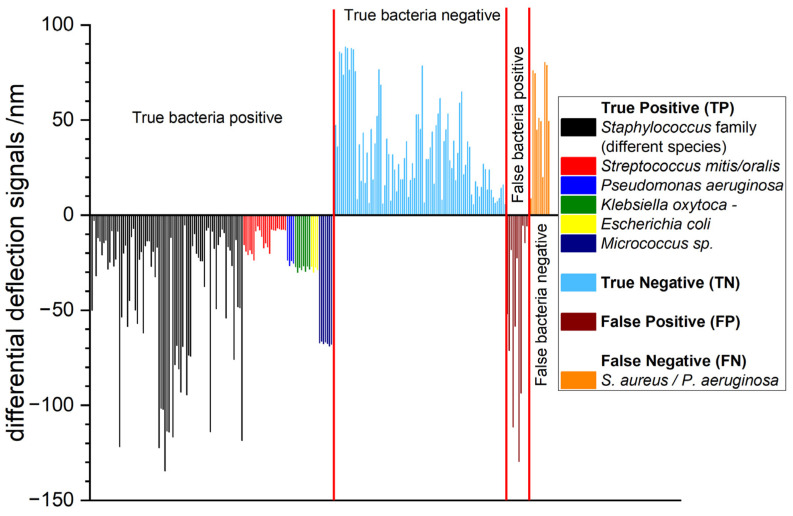
Measurements of total RNA samples from pediatric patients depicting true bacteria positive, true bacteria negative, false bacteria positive and false bacteria negative results. True bacteria positive findings are categorized by different bacterial species (shown in different colors). Healthy individuals can be recognized by a positive differential signal whereas diseased pediatric patients exhibit a signal with a negative sign.

Sepsis in pediatric patients covered in this study was caused by a wide variety of bacterial species. In Figure 8 the absence or presence of a bacterial infection can be clearly inferred by the sign of the differential deflection signal in the vast majority of cases. The low variation of response magnitudes reflects the high reproducibility of the approach. Only a few measurements show false negative or false positive results as backed up by clinical culturing findings. False positive results can be explained by the superior sensitivity of nanomechanical biosensors compared to standard culturing techniques. Standard clinical culturing techniques seem not to be sensitive enough to detect minute bacterial infections. False negative result may originate from possible contaminations during the culturing process.

**Figure 9:**
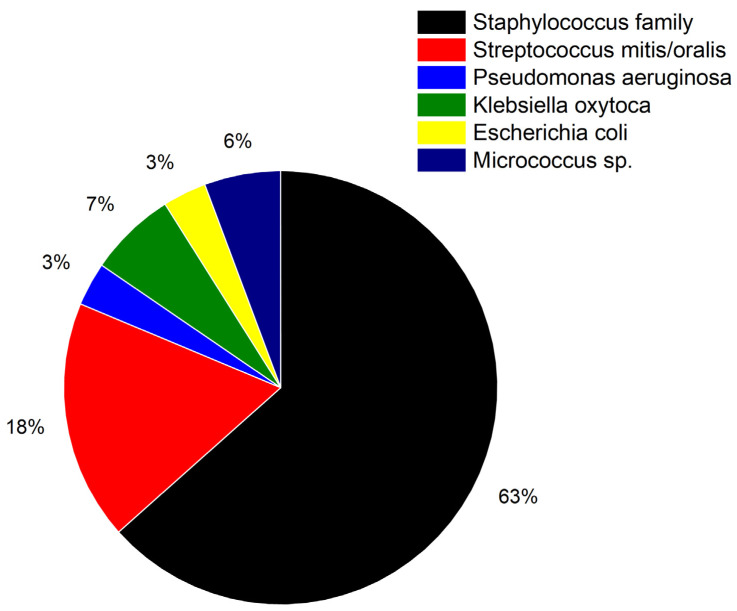
Pie chart showing the distribution of bacterial species covered in this study. The colors match the colors in Figure 8. Determination of species was accomplished by previous culturing.

In Figure 9 the percentage of different bacterial species is shown. Characterization showed the presence of gram negative (*P. aeruginosa*, *K. oxytoca* and *E. coli*, total ca. 13%) as well as gram positive (*Staphylococcus* family, *S. mitis*, *S. oralis* and *Micrococcus* species ca. 87%) bacterial species.

## 4. Discussion

Compared to other methods nanomechanical sensors show a higher sensitivity and a faster measurement time. Particularly the standard culturing technique shows a lower sensitivity and slower diagnostic time as shown in Table 2.

Higher SP and PPV values obtained with the culturing technique can be explained by the fact that as soon as bacteria have grown the species can be readily identified. Higher PPV values can be explained because false positives cannot occur in culturing. The low NPV results probably from a lack of sensitivity of the culturing method.

Bacteria detection using nanomechanical recognition of 16S-rRNA is a recent very powerful method, but should be discussed in comparison with more traditional methods being applied currently in standard laboratory and clinical routine. Among the most frequently used methods in clinics are matrix-assisted desorption/ionisation time-of-flight mass spectrometry (MALDI-TOF) [49,50,51,52], fluorescence-based sensors [53,54], colorimetric sensors [55] and Surface-Enhanced Raman-Scattering [56]. Further concepts include voltammetric sensors [57], specific ligands for label-free detection of whole bacteria [58]. Bacteriophages are viruses that infect bacteria and can be easily produced in large amounts. Detection is often done using electrochemical of optical techniques, such as surface-enhanced Raman spectroscopy [59]. The use of bacteria-imprinted polymers [60] takes advantage of mimicking bacterial structure by providing a template fabricated by lithographic, sol-gel or polypyrrole imprinting. Recognition of bacteria is performed by electrochemistry, SPR, colorimetric or fluorescence techniques. Methods based on bioreceptors [61] are divided into affinity-type sensors based on nucleic acid probes/antibodies and catalytic-type sensors which utilize molecules that bind analytes via enzymes (as in ELISA), cells or tissues. Another promising strategy is the use of aptamers to detect bacteria [62,63,64]. Aptamer sensors can be based on fluorescence probes, colorimetric assays, molecules that bind to aptamers and excite surface plasmon waves upon incidence of light, colour change responses-based surface reactions in lateral flow assays, as well as electrochemical reactions, i.e., changes in current, voltage or impedance. Both detection of bacteria in liquid as well as airborne bacteria has been achieved [62]. Often, whole libraries of aptamers (SELEX, systematic evolution of ligands by exponential enrichment) are used for detection of a wide range of bacteria based on single-stranded oligonucleotides [63].

Table 3 summarizes the limit of detection (LOD) for different recently introduced bacteria reporter assays. Sensitivity among the various techniques varies from 5–6 CFU/mL (nanomechanical biosensors, SERS dual-mode aptasensor) to 21,000 CFU/mL (ELISA).

## 5. Conclusions

This work highlights the feasibility of the method to diagnose sepsis in pediatric patients. Rapid identification of sepsis causing bacterial pathogen is vital as sepsis is a life-threatening condition. It can result in inflammatory responses and organ failure if not treated properly. Time consuming culturing procedures are still the gold standard, which can take up to 72 h. Our approach based on nanomechanical sensor arrays provides ultra-rapid and high sensitivity.

We detect binding of 16S-rRNA to matching oligonucleotide probes immobilized on nanomechanical biosensors to reliably identify bacterial infections within minutes. A study encompassing 233 samples from pediatric patients shows that bacteria positive (diseased) patients reveal negative differential signal and bacteria negative (healthy) patients exhibit a positive differential signal. Different bacterial species do not cause any problems for rapid diagnosis of sepsis. The high sensitivity and prompt response make our technique superior to classical bacterial culturing. The general applicability of the 16S-rRNA probe enables an amplification and label free assay. We envisage our method to be a significant input to rapid, early and sensitive diagnosis from the bench to the bedside, directly from patients’ blood without requirements for cultivation.

## Figures and Tables

**Table 1:** Oligonucleotide probes used in the study.

Probe	Sequence	Use
16S-rRNA	^5′^GGACTACCAGGGTATCTAAT^3‘^	Bacteria detection using 16S-rRNA [44].
polyAC	^5′^ACACACACACACACACACAC^3’^	Reference sequence for non-specific binding [45].

**Table 2:** Calculated values for Sensitivity (SE), Specificity (SP), Positive Predictive value (PPV), and Negative Predictive Value (NPV).

Assay	SE	SP	PPV	NPV	Diagnostic Time
**Nanomechanical Sensors**	**93%**	**88%**	**92%**	**89%**	**1 h**
ELISA	82%	73%	65%	88%	24 h
RT-PCR	77%	88%	69%	91%	6 h
Culture	60%	100%	100%	62%	Up to 3 days

**Table 3:** Comparison of reporter assays adapted from [64] with added data from nanomechanical microcantilever assay.

Assay	Limit of Detection (LOD)
Nanomechanical Sensors [10]	5 CFU/mL
Immunomagnetic quantum dots	1.0 × 10^3^ CFU/mL
ELISA	2.1 × 10^4^ CFU/mL
qPCR	125 CFU/mL
QCM sensor	150 CFU/mL
Colorimetric aptasensor	100 CFU/mL
Electrochemical impedimetric immunosensor	1.0 × 10^3^ CFU/mL
Colorimetric and SERS immunochromatographic assay	50 CFU/mL
Dual-mode aptasensor	6 CFU/mL

## Data Availability

The data presented in this study are available on reasonable request from the corresponding authors.
